# The 3' region of Human Papillomavirus type 16 early mRNAs decrease expression

**DOI:** 10.1186/1471-2334-5-83

**Published:** 2005-10-14

**Authors:** Jeppe Vinther, Maiken W Rosenstierne, Karen Kristiansen, Bodil Norrild

**Affiliations:** 1 Institute of Molecular Pathology, University of Copenhagen, Blegdamsvej 3C, Bldg. 6.2, DK-2200 Copenhagen N, Denmark; 2 Institute of Molecular Biology, University of Copenhagen, Universitetsparken 15, Bldg. 10, DK-2100 Copenhagen Ø, Denmark; 3 BioCentrum-DTU, Technical University of Denmark, Building 208, DK-2800 Lyngby, Denmark

## Abstract

**Background:**

High risk human papillomavirus (HR-HPV) infects mucosal surfaces and HR-HPV infection is required for development of cervical cancer. Accordingly, enforced expression of the early HR-HPV proteins can induce immortalisation of human cells. In most cervical cancers and cervical cancer cell lines the HR-HPV double stranded DNA genome has been integrated into the host cell genome.

**Methods:**

We have used a retroviral GUS reporter system to generate pools of stably transfected HaCaT and SiHa cells. The HPV-16 early sequences that are deleted upon integration of the HPV-16 genome was inserted into the 3' UTR of the reporter mRNA. Pools containing thousands of independent integrations were tested for the steady state levels of the reporter mRNA by Real Time PCR and reporter protein by a GUS enzymatic activity assays. In addition, we tested the cellular distribution and half lives of the reporter mRNAs. The integrity of the reporter mRNAs were tested by northern blotting.

**Results:**

We show that the 3' region of the HPV-16 early mRNAs (HPV-16 nucleotide (nt.) 2582–4214) act in cis to decrease both mRNA and protein levels. This region seems to affect transcription from the exogenous minimal CMV promoter or processing of the reporter mRNA. The observed repression was most pronounced at the protein level, suggesting that this sequence may also affect translation. For the HPV types: 2, 6, 11, 13, 18, 30, 31, and 35 we have investigated the regulatory effect of the regions corresponding to the HPV-16 nt. 3358–4214. For all types, except HPV-18, the region was found to repress expression by posttranscriptional mechanisms.

**Conclusion:**

We find that the 3' region of HPV-16 early mRNAs interfere with gene expression. It is therefore possible that the deletion of the 3' part of early HPV-16 mRNAs occurring during cervical oncogenesis could contribute to transformation of cells through deregulation of the viral oncogene synthesis. Moreover, we find that the corresponding region from several other HPV types also repress expression, suggesting that the repression by this region may be a general feature of the HPV life cycle.

## Background

**Figure 1:**
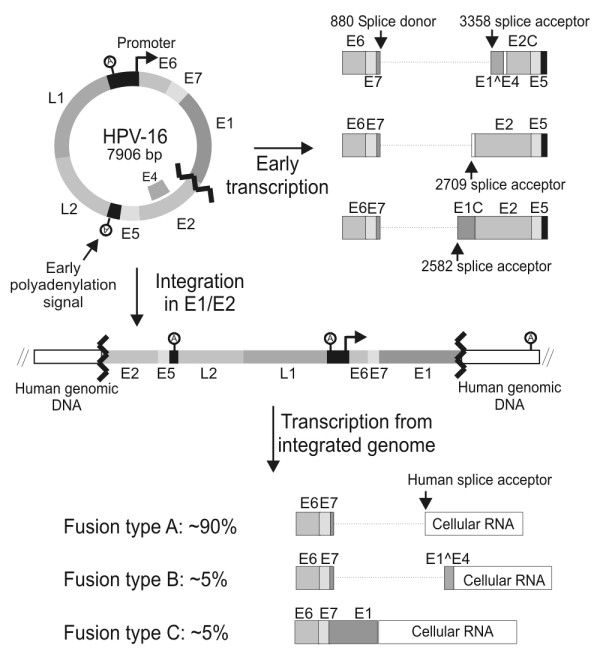
**HPV-16 integrations during oncogenesis**. In normal HPV-16 infections the HPV-16 genome is present as an episome. The mRNAs that encode the HPV-16 E6 and E7 oncogenes are transcribed by the main promoter, spliced from the 880 splice donor and uses the early polyadenylation signal. During cervical oncogenesis the HPV-16 genome is integrated into the cellular genome. This integration occurs in the E1/E2 region and leads to generation of chimeric mRNAs that encode E6 and E7. Most often the chimeric mRNAs are of the A type that are spliced to a cellular splice acceptor [14].

Human Papillomaviruses (HPV) form a large family of homologous circular double stranded DNA viruses that infect the cutaneous and mucosal surfaces of humans. Genital infections with HPV are sexually transmitted and occur frequently in women. Although the majority of infected women will clear the infection within a relatively short time period, some develop a persistent infection [1,2]. This initial clearance may depend on immunological or non-immunological mechanisms [3]. The genital HPV types can be divided into high and low risk types. The high risk HPV (HR-HPV) types (16, 18, 31, 33, 35, 39, 45, 51, 52, 56, 58, 59, 68, 73, and 82) are classified as carcinogenic to humans [4], because epidemiological and molecular studies have shown that infection with one of these HR-HPV types is necessary, but not sufficient for development of cervical cancer [5]. Only a small fraction of persistent HR-HPV infections will progress to high grade cervical lesions and cervical carcinoma. At the molecular level the development of cervical cancer is driven by enforced expression of two oncoproteins (E6 and E7) encoded by the HR-HPV types [6]. HR-HPV E6 and E7 target a large number of cellular proteins involved in normal cellular regulation and in this way facilitate cellular and viral DNA replication in differentiating post mitotic keratinocytes [7,8]. This is crucial for amplification of the viral genome and completion of the viral lifecycle. In the normal viral lifecycle E6 and E7 are expressed at low levels and preferably in the differentiating cells. Probably the low expression of the viral genes helps the virus to avoid detection by the host immune system. The regulation of E6 and E7 expression is complex and occurs on many levels. Interestingly, in most cervical cancers and cervical cancer cell lines the HR-HPV double stranded DNA genome has been integrated into the host cell genome [9-12]. There seems to be little or no preference for integration in specific genes, which suggests that insertional mutagenesis generally is not involved in the generation of cervical cancer cells [13-15]. Conversely, investigations have shown that in a high percentage of cervical cancers and cell lines derived from cervical cancers the circular viral genome has been linearized in the sequence that encode the HPV E1 and E2 proteins before integration (figure 1) [9,14,16,17]. These findings demonstrate that integration in this exact region of the HPV genome confers a selective advantage to cells during cervical oncogenesis and strongly suggest that this region could be of major importance for regulation of the E6 and E7 oncogenes during the normal viral lifecycle. Many studies have addressed the nature of this selective advantage, and several different mechanisms appear to be involved. First, breakage and integration of the HPV genome in E1 or E2 will eliminate expression of the viral proteins E1, E2, E5, L1 and L2. The elimination of E2 expression is thought to be of particular importance for malignant progression. It has been shown that the HPV promoter region contains four E2 binding sites and binding of HPV E2 proteins to these sites repress viral transcription [18,19]. Therefore abrogation of the E2 expression probably leads to increased transcription of E6 and E7. Also, the HR-HPV E2 proteins can induce apoptosis independently of other HPV proteins [20,21]. Furthermore, it is possible that the loss of HPV protein expression facilitates evasion of the immune system, because many women have immunoreactivity towards E2 epitopes that are conserved between high and low risk HPV types [22]. Second, integration may have a direct effect on transcription from the HPV promoter. It is possible that regulatory elements present in the cellular sequence adjacent to the integrated viral sequence stimulate transcription from the viral promoter. Also several Matrix Attachment Regions (MARs) are present in the HPV-16 genome and it has been found that the HPV-16 MARs repress transcription from a reporter construct when in the episomal form. However, when an identical construct was stably integrated into the cellular genome the same regions were found to stimulate transcription [23]. Third, as a consequence of integration the 3' part of the HPV mRNA will be deleted and replaced with sequences derived from the host cell (see figure 1). Thus, in these cells E6 and E7 will be expressed from a chimeric HPV/cellular mRNA. It has been shown that such chimeric mRNAs are present at higher steady state levels than the normal HPV mRNAs [17]. Expression of the E6 and E7 oncogenes from the integrated HPV genome requires that a polyadenylation signal located in the host cell DNA is used for processing of the chimeric mRNA. Investigations have shown that the HPV-31 polyadenylation signal functions quite inefficiently [24] and usage of an efficient cellular polyadenylation signal may therefore enhance the expression of the E6 and E7 oncogenes. Another possibility is that the stability or the translation of the chimeric mRNA is enhanced compared to the normal HPV mRNA. It has been demonstrated that the A/U rich 4005–4213 region from HPV-16 decreased the half-life of a reporter messenger, when inserted downstream of β-globin reporter construct [17]. In another study the same region was shown to decrease the steady state level of the early HPV mRNA by 1.6 fold [25]. This indicates that the expression of E6 and E7 may be enhanced through the deletion of the U rich region.

All HPV-16 mRNAs that encode E6 and E7 contain the sequence from the splice acceptor located at nt. 3358 to the early polyadenylation signal located at nt. 4215 and this sequence may be involved in regulation of the HPV-16 E6 and E7 expression in the normal HPV life cycle. Furthermore, the large majority of chimeric mRNAs created by integration of the HPV-16 genome in cervical cancer cells are spliced from the 880 splice donor located shortly downstream of the E7 reading frame to a cellular splice acceptor (figure 1) [14]. Thus, the elimination of the HPV-16 sequence from nt. 3358–4214 may also be important for regulation of E6 and E7 during the normal HPV-16 lifecycle and could contribute to selection of cells containing integrated HPV-16 genomes during cervical oncogenesis. Here, we demonstrate that the HPV sequence downstream of the HPV-16 nt. 2582 and 3358 splicesites decrease mRNA steady state level and may also affect mRNA translation. Homologous sequences from other HPV types were also found to decrease gene expression.

## Methods

### Cloning of reporter construct

The HRSp-GUS retroviral reporter vector [26,27] was generously provided by professor Helen Blau, Stanford University School of Medicine, California. HPV sequences were PCR-amplified from plasmid templates (pBR322-HPV-2, pSV010-HPV-6, pBR322-HPV-11, pBR322-HPV-13, pBR322-HPV-16, pBR322-HPV-18, pBR322-HPV-30, pBR322-HPV-31 and pBR322-HPV-35 (short BamHI fragment)) with *pfu *polymerase (Promega) and primers from MWG Biotech, Ebersberg, Germany. The following regions were amplified : HPV16: 2582–4214, 3358–4214, 4063–4214, 4063–4310, HPV-18: 3378–4234, HPV-31: 3295–4137, HPV-6: 3325–4377, HPV-11: 3325–4370, HPV-35: 3301–4158, HPV-2: 3299–4387, HPV-13: 3332–4331, HPV-30: 3206–4216. Primers included restriction sites for cloning into Apa I / PinA I digested pHRSp-GUS or into Xho I / PinA I digested pHRSp-GUS (HPV-16 nt. 4063–4310). Plasmid DNA for transfection was prepared with Qiagen kits and all inserts were sequenced.

### Cell culture and retroviral infections

The SiHa cell line was obtained from American Type Culture Collection (ATCC number HTB-35) and professor Norbert Fusenig, DKFZ, Heidelberg, kindly provided the HaCaT cell line. The PG13 amphotropic packaging cell line was a gift from PhD Lene Pedersen, Aarhus University, Denmark. 8,6 cm dishes with PG13 cells (grown in 10% neonatal calf serum) were transfected with pHRSp-GUS reporter constructs using the LipofectaminePLUS reagent (Invitrogen). 72 hours post transfection Puromycin (Sigma P7255) was added to a final concentration of 2,5 μg/ml for selection of stably transfected cells. The cells were transferred to T175 flasks that were allowed to grow to confluence. Viral supernatants were harvested by changing the media to 10 % Foetal calf serum (Invitrogen) and incubating the cells for 24 hours at 32°C, 5% CO2. The supernatants were filtered through a 0,45 μm syringe filter (Satorius) and Polybrene was added to a final concentration of 8 μg/ml. For each construct three 8.6 cm dishes of HaCaT or SiHa were each infected 4 hours with 4 ml of viral supernatant. 72 hours after transduction the infected cells were selected by adding 2,5 μg/ml Puromycin (Sigma P7255) to the cell media. The number of colonies was evaluated for each infection (pool), so that at least 1000 different colonies were present in each pool. For each construct three independent infections were performed.

### Quantification of mRNA levels by Real time RT-PCR

1 × 10^6 ^HaCaT or SiHa cells from the different cell pools (three for each construct) were plated in 8.6 cm cell culture dishes and after 48 hours total RNA was isolated with the Qiagen RNeasy kit according to the instructions of the manufacturer. 10 μg RNA were treated with Amplification grade RNase free DNase I (Invitrogen). Real Time PCR reactions were run on a MJ Research DNA Engine Opticon™. Primers pairs specific for the GUS (5'-GTGATGATAATCGGCTGATG-3' and 5'-CCTGCGTCAATGTAATGTTC-3') and PURO (5'-AGGGCAAGGGTCTGGGCA-3' and 5'-TCGGCGGTGACGGTGAAG-3') coding sequences were designed. The GUS primer set was used with an annealing temperature of 58°C and the PURO primer set with an annealing temperature at 63°C. The QuantiTect SYBR Green RT-PCR Kit from Qiagen was used to assemble 25 μl RT-PCR/PCR reactions in triplicate. The following PCR profile was used: 30 min. at 50°C, 15 min. at 95°C, (15 s at 94°C, 30 s at 58 or 63°C, 1 min at 72°C) × 40 followed by recording of a melting curve. The threshold cycle Ct for individual reactions was determined with the Opticon software. All templates were checked for DNA contamination by omitting the reverse transcriptase in control reactions. The GUS mRNA level relative to the PURO mRNA level for each construct was calculated with the 2^-ΔΔCt ^method and expressed relative to the empty vector [28]. The GUS mRNA levels were normalized to the PURO mRNA levels to ensure that differences between constructs in the efficiency of retroviral infection does not influence the results [27]. ΔC_t _values for individual pools were calculated and the ΔC_t _average for the empty vector was treated as an arbitrary constant and used to calculate ΔΔC_t _values for all pools. The mRNA levels for individual pools were calculated by evaluating the 2^-ΔΔCt ^term. The results for the three independent pools for each construct were averaged and the standard error of the mean was calculated. For mRNA half-life experiments Actinomycin D (Sigma A9415) was added to the medium to a concentration of 2 μg/ml and total RNA was harvested from the cells at t = 0, 1, 3, 6, 9, 15 hrs. Aliquots containing equal amounts of total RNA were used as templates for Real Time PCR analysis with the GUS specific primerset. The GUS mRNA levels for each timepoint were calculated from the C_t _values, averaged and expressed relative to the mRNA level at the time of Actinomycin D addition. For examination of nuclear/cytoplasmic mRNA ratios SiHa cells were trypsinised and 2 × 10^6 ^cells were used for preparation of cytoplasmic RNA with the RNeasy kit from Qiagen. The nuclear pellets were washed three times in RLN buffer (Qiagen) and used for isolation of nuclear RNA with the Qiagen RNeasy procedure for total RNA. The mRNA levels were determined with Real time PCR. The GAPDH mRNA was detected with the following primer set (5'-GAAGGTGAAGGTCGGAGTC-3') and (5'-GAAGATGGTGATGGGATTTC-3').

### Northern blot analysis

Total RNA (app. 7,5 μg) from the SiHa cell pools stably transfected with the different constructs was separated on a MOPS 1% agarose gel, transferred to a BrightStar Membrane (Ambion) and crosslinked with the autocrosslink setting in a Stratalinker (Stratagene) according to the recommendations in the NorthernMax kit from Ambion. Nt. 6–131 and nt. 538–1037 of the GUS reporter mRNA were cloned into pBluescript II SK (+) and used for preparation of radioactively labelled probes by in vitro transcription with Radioactive labelled UTP [α-^32^P, 800 Ci/mmol] and the T7 Maxiscript kit (Ambion) according to the recommendations of supplier. The crosslinked membrane was hybridised over night with 1 × 10^6 ^cpm probe per ml of ULTRAhyp solution at 72°C and washed the following day according to the instructions in the NorthernMax protocol. Finally, the membrane was exposed to Biomax MR film (Kodax) for 72 hours at 4°C.

### Quantification of total protein and GUS protein

1 × 10^6 ^HaCaT or SiHa cells from the different cell pools (three for each construct) were plated in 8.6 cm cell culture dishes. After 48 hours cells were scraped in lysis buffer: 100 mM potassiumphosphate (pH 7.8) and 0.2 % Triton X-100 with DTT added to 0,5 mM immediately before use. Membrane debris was pelleted by centrifugation (15000 g, 4°C) for 3 min. and the supernatant was collected and stored at -80°C. The Coomassie^®^Plus Reagent (PIERCE) was used to determine the total protein concentration of the extracts. The level of GUS protein in the extracts was determined with the FluorAceTM β-glucuronidase Reporter Assay kit from BIORAD. All cell extracts were diluted 1:10, 1:100, 1:1000 or 1:10000 to ensure that the measurements were within the linear range of the assay. Fluorescence was counted on a Wallac VICTOR2 Multilabel Counter. The GUS protein levels were normalized with the total protein levels and expressed relative to the HRSp-GUS vector. For each construct the three independent pools were averaged and the standard error of the mean was calculated. Translational was evaluated for the different constructs by normalizing the GUS protein levels with the total levels of the GUS mRNA (not normalized to PURO) as determined by Real Time RT-PCR.

## Results

**Figure 2:**
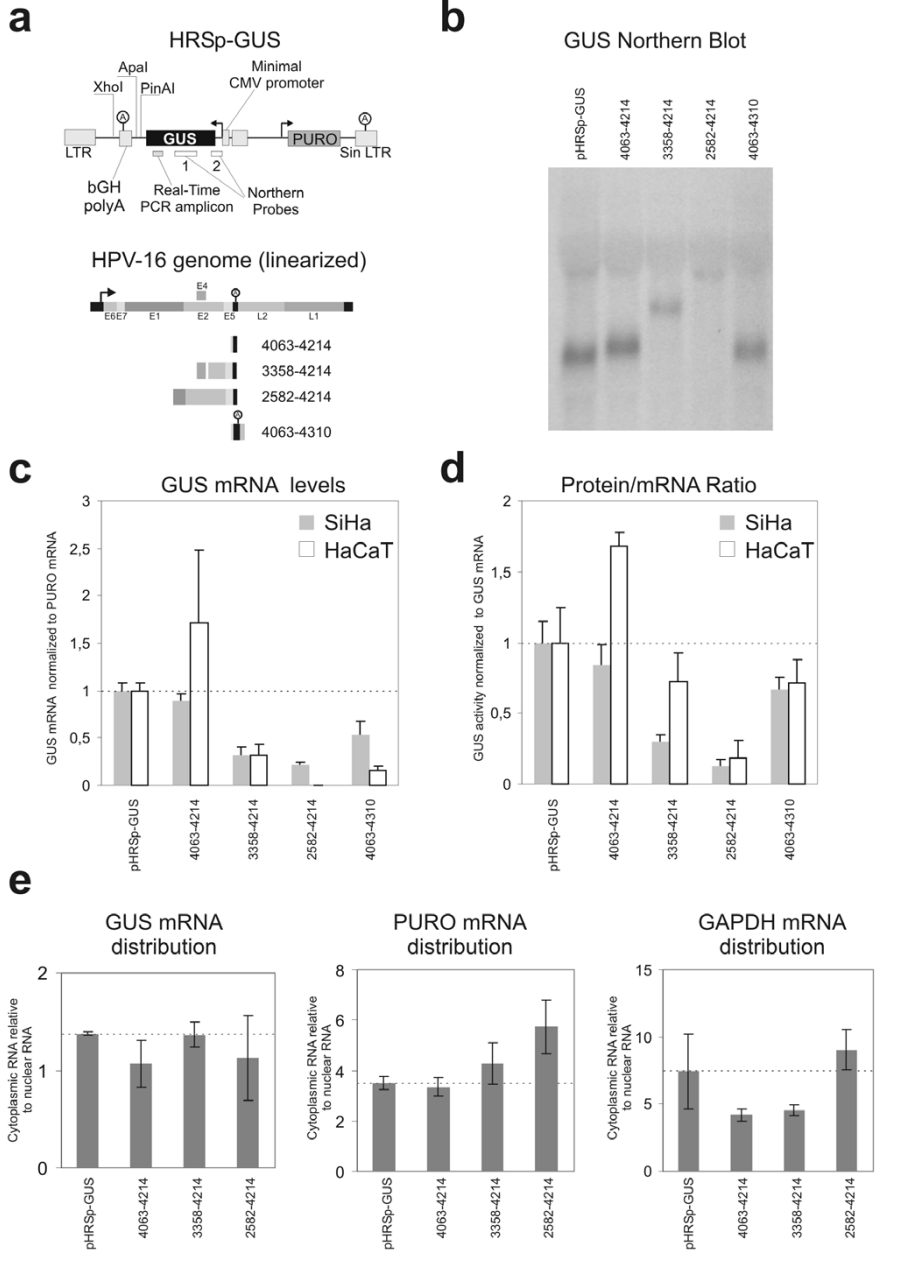
**Effects of HPV-16 sequences on the steady state level of GUS mRNA and GUS protein**. *a*) Schematic representation of the HRSp-GUS retroviral reporter vector [26,27] and the location of the GUS northern probes and the Real Time PCR amplicon. *b*) Northern Blot analysis of mRNA from SiHa cell pools (only GUS probe 1 experiment is shown). Blotting of mRNA from the HaCaT cell pools is not shown, but yielded similar results. *c*) GUS mRNA steady state levels. For each construct the steady state levels of the GUS mRNA and PURO mRNA were determined with real-time RT-PCR. The GUS mRNA levels were normalized with the PURO mRNA. The data plotted is the average of results from three independent pools of infected SiHa or HaCaT cells expressed relative to the empty HRSp-GUS construct. The error bars indicate the standard error of the mean. *d*) Reporter protein produced per reporter mRNA. For each construct the steady state GUS activity and total protein levels were determined. The GUS activities were normalized with the total protein levels. Protein/RNA ratios of the different reporter mRNA was evaluated by calculation of the ratio between the steady state levels of GUS protein to GUS mRNAs (not normalized for PURO) The data plotted is the average of results from three independent pools of infected SiHa or HaCaT cells expressed relative to the empty HRSp-GUS construct. The error bars indicate the standard error of the mean. *e) *Cellular distribution of Reporter mRNAs. The ratio of cytoplasmic to nuclear RNA for the GUS mRNA (left), PURO mRNA (middle) and GAPDH mRNA (right) in the different reporter cell lines.

We have investigated the effect of posttranscriptional regulation, including translation, on the expression of the HPV-16 early genes with the HRSp-GUS vector that has been designed for investigation of the regulatory properties of UTR sequences [26,27] (see figure 2a). The main advantage of this vector is that the reporter mRNAs are expressed at low levels from one or a few retroviral integrations. Expression from integrated reporter constructs is a good model for the integrated oncogenic HPV DNA that is found in advanced cervical lesions and furthermore the low expression ensures that transacting factors are not titrated out. In the reporter vector we have inserted different HPV-16 sequences 3' to the GUS coding sequence to determine how they influence the steady state level of the reporter mRNA and reporter protein (figure 2a). All steady state levels were determined as the average of data collected from three independent cell pools each containing several thousand clones of stably transfected SiHa and HaCaT cells. Total RNA from different clones was used for Northern Blot analysis with two probes specific for the GUS sequence. One of these probes anneals to the first 120 bases of the reporter mRNA to ensure that all spliced reporter as well as the full-length mRNAs would be detected. The probes only detected the expected full-length mRNAs and no shorter spliced forms. For the nt. 2582–4214 reporter construct no mRNA was detectable (figure 2b). We find that the inclusion of the A/U rich HPV-16 sequence from nt. 4063 to 4214 in the reporter construct has little inhibitory effect on steady state level of the reporter mRNA (figure 2c). In the SiHa cell line the reporter mRNA is expressed to app. 90 % of the level of the empty vector, whereas the HPV sequence seems to have a stabilizing effect in the HaCaT cell line, although we have large differences between our pools for this reporter construct. The region from the HPV-16 nt. 3358 splice acceptor to the early polyadenylation signal is present in all HPV-16 mRNAs that encode E6 and E7. We find that HPV-16 nt. 3358–4214 region shows a significant repression of the steady state level mRNA both in the SiHa and HaCaT cell line when inserted into the HRSp-GUS vector (figure 2b). The HPV-16 genome also contains splice acceptors at nucleotides 2582 and 2709 and we therefore investigated the steady state level of a construct with the HPV-16 nt. 2582–4214 sequence inserted. In the SiHa cell line this construct further decreased the steady state level of the reporter mRNA and in the HaCaT cell line only extremely low levels of GUS mRNA were detected (figure 2b and 2c).

It has previously been demonstrated that the HPV-31 early polyadenylation signal functions inefficiently [24] and we therefore made a construct with the HPV-16 early polyadenylation region included instead of the bovine Growth Hormone (bGH) polyA region present in the HRSp-GUS vector (figure 2a). In our experiments usage of the HPV-16 early polyadenylation signal (HPV-16 nt. 4063–4310) does not significantly reduce the steady state levels of the reporter mRNA compared to the similar construct containing the bGH polyadenylation region (HPV-16 nt. 4063–4214) (figure 2b). This indicates that in SiHa and HaCaT keratinocytes the HPV-16 early polyadenylation signal functions reasonably efficiently.

Although it is quite possible that the expression of the HPV-16 E6 and E7 proteins is regulated at the protein level by the sequences downstream of the reading frames, this has to our knowledge never been investigated in any detail. We determined the steady state levels of the GUS reporter protein normalized to total protein levels for the different constructs. The normalized protein levels reflect the full regulatory influence of the inserted sequences, including effects on mRNA processing, mRNA transport, mRNA stability and translation. To assess the influence that the inserted HPV sequences have on the amount of protein produced per mRNA we calculated the ratio between the steady state levels of GUS protein (normalized to total protein) and the steady state levels of the GUS mRNA (normalized to total RNA)(figure 2d). This normalization reveals that in neither cell line the HPV-16 nt. 4063–4214 region changes the protein/mRNA ratio, whereas the HPV-16 nt. 2582–4214 region greatly reduces the protein/mRNA ratio. The HPV-16 nt. 3358–4214 region significantly reduces the protein/mRNA ratio in the SiHa cell line, but not in HaCaT cells. Inclusion of the HPV-16 polyadenylation region instead of the bGH polyadenylation region in the HPV-16 4063–4310 construct should give mRNAs with native HPV-16 3' ends. Compared with the similar mRNA that contains the bGH polyadenylation region (HPV-16 nt. 4063–4214), this mRNA produces less protein in the HaCaT cell line, whereas no significant difference is present in SiHa cells (figure 2d). Only the cytoplasmic pool of mRNA is available for translation and it is therefore possible that the reduction in protein/mRNA ratio that we observe is caused by an altered distribution of the reporter mRNAs. We therefore isolated cytoplasmic and nuclear mRNA fractions and determined the mRNA concentrations by real time PCR. None of the inserted HPV-16 sequences affect the distribution of the GUS reporter mRNA or the PURO and GAPDH controls (figure 2e).

**Figure 3:**
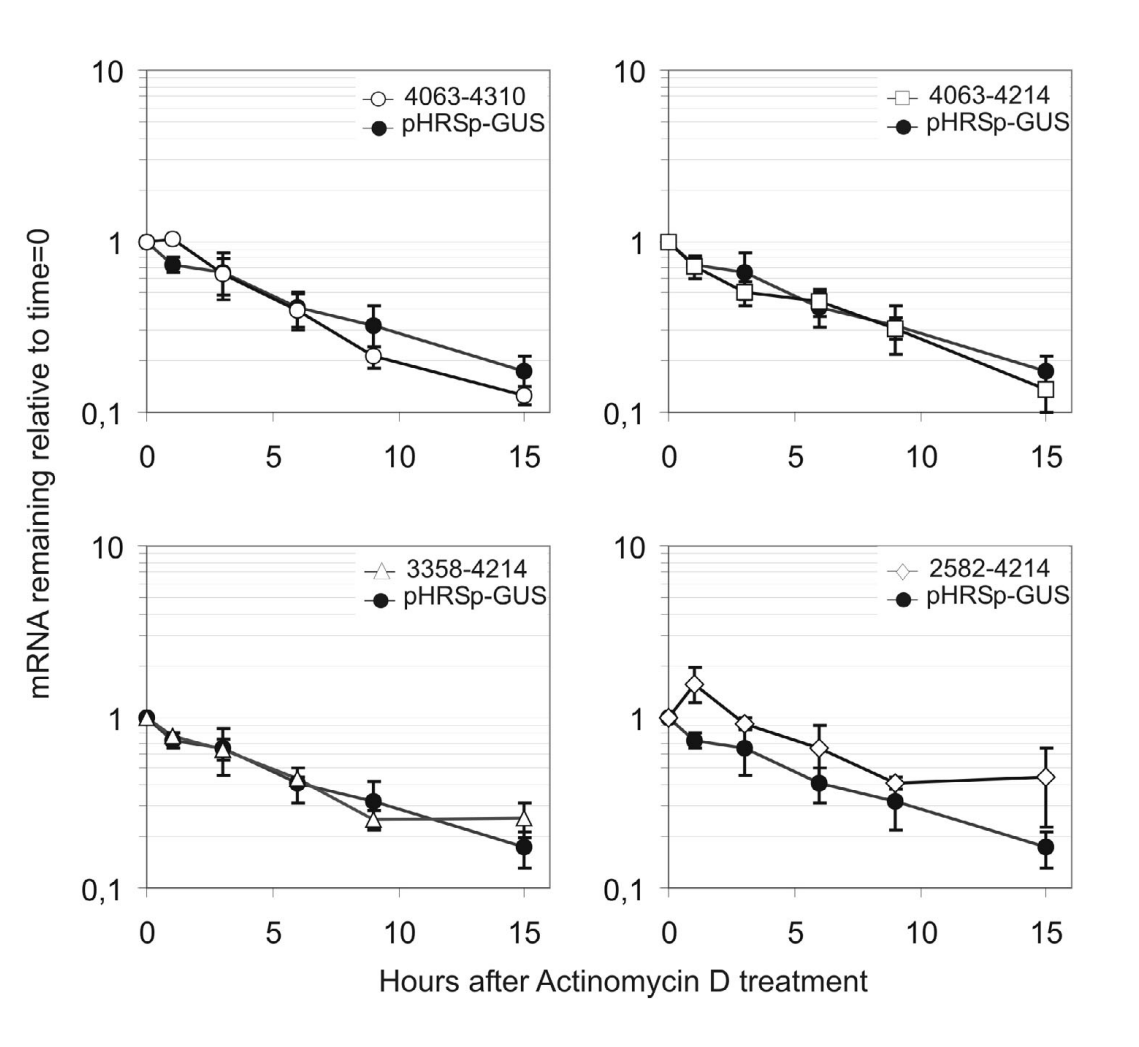
**Decay of reporter mRNAs**. SiHa cells expressing the indicated constructs were treated with Actinomycin D to inhibit transcription. mRNA levels of the different GUS reporter mRNAs normalized to total RNA was determined with real-time RT-PCR at the indicated time points. At least three different measurements were made for each timepoint and the data plotted is the average relative to the mRNA level at the time of Actinomycin D addition. The error bars indicate the standard deviation.

The effects that we observe on mRNA steady state levels for the nt. 3358–4214 and 2583–4214 constructs could be the result of decreased stability of the reporter mRNAs or decreased transcription. To differentiate between these two possible mechanisms, we determined the half-lives of the two reporter mRNAs (figure 3b). The nt. 3358–4214 and 2583–4214 reporter mRNAs did not have significantly lower half-life than the nt. 4063–4214 and control mRNAs, which indicates the reduced steady state level is caused by inhibition of transcription.

**Figure 4:**
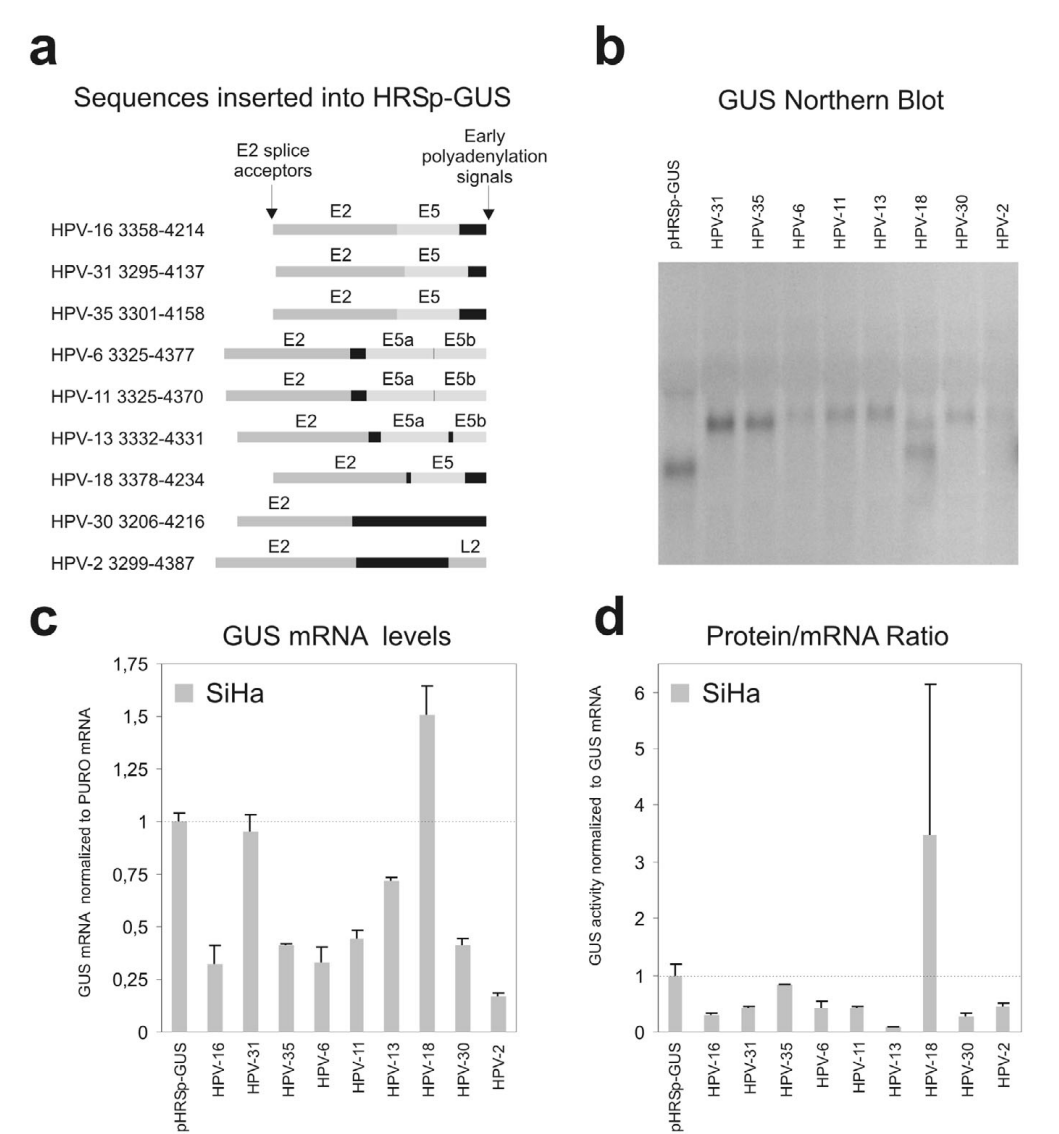
**Effects of HPV 3' early regions on the steady state level of GUS mRNA and GUS protein**. *a*) Schematic representation of the HPV sequences that were cloned into the retroviral HRSp-GUS reporter vector [26,27]. *b*) Northern Blot analysis of mRNA from SiHa cell pools (only GUS probe 1 experiments shown). *c*) GUS mRNA steady state levels. For each construct the steady state levels of the GUS mRNA and PURO mRNA were determined with real-time RT-PCR. The GUS mRNA levels were normalized with the PURO mRNA. The data plotted is the average of results from three independent pools of infected SiHa cells expressed relative to the empty HRSp-GUS construct. The error bars indicate the standard error of the mean. *d*) Protein produced per reporter mRNA For each construct the steady state GUS activity and total protein levels were determined. The GUS activities were normalized with the total protein levels. The protein/RNA ratios of the different reporter mRNA was evaluated by calculation of the ratio between the steady state levels of GUS protein to GUS mRNAs (not normalized for PURO). The data plotted is the average of results from three independent pools of infected SiHa cells expressed relative to the empty HRSp-GUS construct. The error bars indicate the standard error of the mean.

The genomic organisation of the different HPV types is highly conserved. They all have two polyadenylation signals that divide the genome into an early and a late region. Moreover all HPV types express E6 an E7 from mRNAs that are spliced to a splice acceptor in the E2 reading frame analogous to the HPV-16 nt. 3358 splice acceptor. We therefore decided to determine if the negative effect on translation that we observe with the HPV16 nt. 3358–4214 region is a general regulatory feature of different HPV types. The region from the E2 splice acceptor to the early polyadenylation signal from HPV types: 2, 6, 11, 13, 16, 18, 30, 31 and 35 was therefore cloned into the 3' UTR of the pHRSp-GUS vector and the steady state level of reporter mRNA and protein was determined after transduction of SiHa cells (figure 4a). To ensure that the reporter constructs expressed the correct reporter mRNA we performed a Northern Blot analysis with both the probes shown in figure 2a. All pools expressed GUS mRNAs of the expected length (figure 4b), except for the HPV-18 construct that expressed an additional, shorter GUS mRNA of unknown structure. We find that all the tested HPV sequences, except the ones derived from 18 and 31, decrease the steady state level of the reporter mRNA compared to the empty vector (figure 4c). The largest repression was observed with the HPV-2 construct. For all types except HPV-35 and HPV-18 the repressive effect of the inserted HPV sequence also decreased the translation (figure 4d). These results therefore indicate that the posttranscriptional repression of the region from the E2 splice site to the early polyadenylation signal is a general feature of different HPV types.

## Discussion

In this study we have investigated if the HPV-16 early genes are regulated at the posttranscriptional level. We find that the HPV-16 nt. 2582–4214 region very efficiently reduce the steady state level of a reporter mRNA and the amount of protein that is produced from the mRNA when compared to the empty reporter vector HRSp-GUS vector (see figure 2). Moreover, a truncated construct containing the HPV-16 nt. 3358–4214 region also decreased the steady state level of reporter mRNA and in one of the two cell lines tested reduced the protein/mRNA ratio. In contrast the HPV-16 nt. 4063–4214 region encompassing the short U rich HPV-16 early 3' UTR only, did not affect expression. The reporter assay that we have used in our experiments is based on observation of steady state levels of the different reporter mRNAs and of reporter protein. It is possible that splice acceptors present in the inserted sequences could stimulate splicing from cryptic splice donors in the reporter sequence and this would make our results very difficult to evaluate. For that reason we used Northern Blot analysis to confirm that all the reporter mRNAs have the expected unspliced structure. All constructs, except the HPV-18 construct, produced only the expected full length unspliced reporter mRNA (figure 2b and 4b). The 2582–4214 construct did not express sufficient amounts of reporter mRNA (full length or spliced) to be detected by Northern Blot. Thus, the lowered steady state levels of reporter mRNA that we observe could reflect a decrease in reporter mRNA half-life, a decreased rate of transcription or inhibition of export of mRNA. Surprisingly, we found that none of our reporter mRNAs containing HPV-16 sequence had a decreased half-life (see figure 3). This indicates that the inserted HPV-16 sequence influence the transcription from the minimal CMV promoter included in the pHRSp-GUS vector or affect the nuclear export of the mRNAs. It is known that HPV-16 E5 region contain a strong matrix attachment region [23,29] and it is possible that this region or other unidentified enhancer elements influence the CMV promoter. Moreover, it is well established that the late mRNAs from several different papillomavirus types contain sequences that interfere with mRNA export [30-33]. Often these sequences bind factors involved in splicing such as U2AF65 [32] and the U1 snRNA [30]. The HPV-16 nt. 3358–4214 and 2582–4214 regions both contain a splice donor located at HPV-16 nt. 3632 and the HPV-16 nt. 2582–4214 region also contain the splice acceptor at HPV-16 nt. 3358. Both these splice sequences diverge from the consensus sequences, but are used during the viral lifecycle and could therefore potentially contribute to the repression that we observe by inhibiting mRNA export and mediation of rapid degradation within the nucleus. Still, we investigated the distribution of the different reporter mRNA (figure 2e) and found that the HPV-16 sequences does not change the distribution of the reporter mRNAs. This suggests that the decrease in steady state mRNA levels is caused by inhibition of the minimal CMV promoter. Alternatively ineffective mRNA processing of the reporter constructs may lead to very rapid degradation (cotranscriptional) of the reporter mRNA that cannot be detected in our RNA half-life experiments.

Jeon and Lambert have previously reported that the very A/U rich 4005–4213 region from HPV-16 very efficiently decreases the stability of a reporter mRNA [17]. The 3' UTRs from short-lived mRNAs often contain destabilizing A/U-rich elements (ARE) [34], but the HPV-16 A/U rich sequence does not contain any of the canonical ARE sequences (AUUUA). AREs and a few other RNA sequences have been found to be highly overrepresented in mRNAs with high turnover rate, but they are not strong predictors of the mRNA turnover rate [35], This emphasizes that the ability of a RNA sequences to destabilize mRNAs is highly context dependent and requires cooperative binding of several factors. In our experiments the HPV-16 sequence from nt. 4063–4214 does not influence the steady state level or decrease the half-life of the reporter mRNA (figure 2a and 3). Schwartz and coworkers did not find any repressive effect of the HPV-16 nt. 4005–4213 region on protein expression in their reporter system [36] and weak inhibitory effect (1.6 fold inhibition) on the mRNA level [25]. It is possible that the upstream nt. 4005–4063 sequence is necessary for the repression observed by Jeon and Lambert and Schwartz and coworkers. Another possibility is that the reporter systems and cell lines used in the different studies influence the regulatory properties of this HPV-16 region.

It has been reported that the early polyadenylation region from HPV-31 is app. 20 fold less efficient than the SV-40 late polyadenylation region and that this difference could be reduced to a 5 fold difference by introduction of an efficient binding site for the cleavage stimulatory factor CstF-64 to the HPV-31 polyadenylation region [24]. Our experiments indicate that the efficiency of the HPV-16 early polyadenylation region from nt. 4215 to 4310 is comparable with the efficiency of the bGH polyadenylation region present in the HRSp-GUS vector (compare 4063–4214 with 4063–4310, figure 2c). Clearly, these findings may reflect a difference in the efficiency of the HPV-16 and HPV-31 early polyadenylation regions or the efficiency of the bGH and SV40 polyadenylation regions. Interestingly, Schwartz and coworkers have recently shown that the U-rich sequence present in the HPV-16 3' UTR enhances polyadenylation from the early HPV-16 polyadenylation signal [25]. The HPV-31 early 3' UTR is less U-rich than the HPV-16 early 3' UTR and may therefore not enhance polyadenylation in the same way. Still, different cells and reporter systems were used in the different studies.

In addition to decreased reporter mRNA steady state levels we also observe that the mRNAs containing the HPV-16 nt. 2582–4214 and nt. 3358–4214 sequences produce less protein per mRNA than the control mRNA. Only cytoplasmic mRNA is available for translation and since we have measured total mRNA concentration, inhibition of mRNA export and accumulation of reporter mRNAs in the nucleus would result in a decreased protein/mRNA ratio in our experiments. We find that the ratio of nuclear to cytoplasmic mRNA is unaltered by the HPV-16 sequences (figure 2e), which indicates that the inserted HPV sequences may influence translation.

A productive HR-HPV infection relies on carefully regulated expression of the HPV gene products. The expression is coupled to the differentiation of the infected cells, so that the viral proteins are expressed at low levels in the basal and suprabasal cell layers, but are expressed at higher levels in the more superficial cells that have exited the cell cycle [37,38]. This strategy most likely helps to keep the HPV infection hidden for the immune system. The repression that we observe by the HPV-16 nt. 3358–4214 and nt. 2582–4214 regions shows that HPV-16 exploits posttranscriptional mechanisms probably to keep the expression of the E6 and E7 proteins at a suitably low level. This is analogous to the repression of the late structural proteins of HPV-16, which also partly occur on the level of mRNA processing, export, stability and translation [30-32,39,40].

We next wondered if posttranscriptional repression could be a general feature of early mRNAs from different HR-HPV types and possibly all HPV types. The HPV types tested include the high risk types HPV-16, HPV-18, HPV-31, and HPV-35 and also the low risk types HPV-6, HPV-11 and HPV-13, which frequently infect the genital tract and cause condylomata acuminata, but are practically absent in high grade lesions and in cervical cancers. In addition, we have tested HPV-2, a benign HPV type with a preference for cutaneous tissue and HPV-30, which is a rather rare HPV type occasionally present in genital infections. We found that the region from the E2 splice acceptor to the early polyadenylation signal from all the tested types except HPV-18 repress expression of the reporter by decreasing mRNA steady state levels or reducing the amount of protein produced or both (figure 4). Like the HPV-16 sequence these HPV sequences therefore contain elements that interfere with transcription and/or processing of the mRNAs to reduce the steady state level of the reporter mRNAs. Moreover, most of them also reduce translation and it is therefore very likely that repression by this region of the HPV genomes in a general feature of the HPV life cycle. The HPV-18 sequence seems to increase expression of the reporter through increased mRNA steady state level and enhanced protein production, but since we find that the HPV-18 construct in addition to the expected full length reporter mRNA also express a shorter mRNA this may be the reason for the increased expression. The finding that the 3' end of most HPV E6 and E7 mRNAs repress expression by posttranscriptional mechanisms is interesting, since it suggests that posttranscriptional repression of the early HPV genes may be a general feature of many HPV types and possibly has evolved to facilitate evasion of the host immune system. Still all the HPV types tested are part of the A supergroup and more distantly related types could be regulated very differently.

## Conclusion

In conclusion, we demonstrate that the HPV-16 nt. 2582–4063 region represses expression at the mRNA and possibly also the protein level. This regulation could be involved in the regulation of the HPV-16 E6 and E7 oncogenes during the normal HPV lifecycle. Also for the HPV types 2, 6, 11, 13, 16, 30, 31, 32 and 35 the region from the E2 splice acceptor to the early polyadenylation signal represses expression of the reporter construct. This indicates that regions downstream of these splicesites function in the HPV lifecycle to repress the expression of the early HPV proteins including the E6 and E7 oncoproteins. For the HR-HPV types deletion of this region through genomic integration occurring in the E1 or E2 open reading frames could contribute to enhanced expression of E6 and E7 and increase the oncogenic potential of the infected cells during cervical oncogenesis.

## Competing interests

The author(s) declare that they have no competing interests.

## Authors' contributions

JV designed the study, carried out cloning, northern blotting and drafted the manuscript. MWR carried out the retroviral infections, Real Time RT-PCR and GUS assays, mRNA half life investigations, fractionations of the cytoplasmic and nuclear mRNAs and helped to draft the manuscript. KK participated in the mRNA half live investigations. BN participated in the design and coordination of the study and helped to draft the manuscript. All authors read and approved the final manuscript.

## Pre-publication history

The pre-publication history for this paper can be accessed here:


